# Optimizing individualized therapy decision-making in multiple myeloma (MM): integration and impact of the Revised Myeloma Comorbidity Index in the MM-tumor board

**DOI:** 10.1007/s00277-024-06010-5

**Published:** 2024-09-21

**Authors:** Esther Dreyling, Gabriele Ihorst, Heike Reinhardt, Jan Räder, Maximilian Holler, Georg Herget, Christine Greil, Ralph Wäsch, Monika Engelhardt

**Affiliations:** 1 Department of Medicine I Hematology and Oncology, Medical Center -University of Freiburg (UKF), Faculty of Medicine, Hugstetterstr. 53, Freiburg im Breisgau, 79106 Germany; 2 Comprehensive Cancer Center Freiburg (CCCF), Medical Center -UKF, Faculty of Medicine, Freiburg im Breisgau, Germany; 3 Clinical Trials Unit, Medical Center -UKF, Faculty of Medicine, Freiburg im Breisgau, Germany; 4 Department of Cardiology and Angiology I, Heart Center, Faculty of Medicine, UKF, Freiburg, Germany; 5 Department of Orthopedics and Trauma Surgery, Medical Faculty, UKF, Freiburg, Germany

**Keywords:** Multiple myeloma (MM), Tumor boards (TBs), Revised Myeloma Comorbidity Index (R-MCI), Therapy adjustment, Geriatric assessment, Frailty

## Abstract

Multiple Myeloma (MM) is a hematological disease predominantly affecting elderly patients. The complexity of current treatment necessitates individualized approaches. Therein, functional assessment (FA) tools, such as the Revised Comorbidity Index (R-MCI) at our University- and Comprehensive Cancer Center Freiburg, play a crucial role. This study aimed to determine (a) the implementation of the R-MCI in our MM-tumor board (MM-TB), (b) its impact on treatment guidance at baseline and (c) potential changes during follow-up. This exploratory study investigated R-MCI coverage and distribution in a cohort of patients with multiple TB presentations. Among them, a follow-up patient cohort undergoing subsequent MM-therapy was analyzed to determine treatment adjustments and changes in patients’ condition measured by R-MCI alterations. During our 3-year assessment period, 565 patients were presented in our MM-TB, totaling 1256 TB-presentations. In the multiple TB presentation cohort, the median number of TB presentations was 3 (range: 2–12). R-MCI scores within the MM-TB were available in 94%, whereas in 6%, the R-MCI had not been integrated. Among these, potential failure to identify the need for treatment modifications was determined. In the follow-up cohort, patient characteristics were typical for referral/university centers. Dose reductions were performed in 55% and were more prevalent among patients with ≥ 4 vs. lesser TB presentations. Most patients (55%) showed a fitness stabilization or improvement via follow-up R-MCI. R-MCI integration in MM-TB exceeded > 90%, indicating its successful integration for treatment support. Our results underscore its value in guiding therapy decisions, providing a comprehensive assessment beyond age considerations.

**Supplementary Information:**

The online version contains supplementary material available at 10.1007/s00277-024-06010-5.

## Introduction

Multiple Myeloma (MM) is a hematological disease that predominantly affects elderly patients. The introduction of proteasome inhibitors (PI), immunomodulatory drugs (IMiDs) and immunotherapeutics (monoclonal and bispecific antibodies [BITEs], antibody drug conjugates [ADC], chimeric antigen receptor [CAR]-T-cells), has significantly enlarged therapeutic options and has improved the prognosis, progression-free- (PFS) and overall survival (OS) of MM patients over the last 20 years [1]. Standard treatment for MM now involves triplet or quadruplet therapy which typically includes a combination of a PI, IMiDs, corticosteroid and monoclonal antibody [2]. Additionally, if patients are deemed fit enough, autologous stem cell transplantation (ASCT) followed by maintenance therapy is considered [2–6].

Due to the availability of numerous therapeutic options, treatment of MM has become more complex in recent years, allowing individualized treatment. It is known for example, that carefully selected older patients can benefit from intensive therapy as much as younger patients. These patients may also receive triplets or quadruplets, tolerate longer treatment sequences, and should be included in clinical trials [2, 7–12]. However, inclusion of elderly patients over the age of 70 years and of vulnerable individuals in clinical trials is notably infrequent. Consequently, determining the eligibility of individuals for intensive and/or novel treatment can be challenging [7–13]. To identify suitable treatment options for these patients, functional assessment (FA) tools have been established [7, 9, 12, 13]. A FA is a multidimensional, multidisciplinary approach to more objectively determine the functional health status of frail, vulnerable and/or elderly patients in order to predict the outcomes and risks of various health conditions and to customize treatment decisions [7–14]. This approach allows for patient selection, reduces risk, and enables the provision of tailored treatments. Medications optimized through FA can minimize severe chemotherapy side effects without compromising treatment efficacy in older cancer patients, leading to improved quality of life (QoL) and fewer unplanned hospitalizations [14–16]. Recently, MM-specific risk scores, such as the International Myeloma Working Group (IMWG)-frailty score, Revised-Myeloma Comorbidity Index (R-MCI), Mayo-risk score, and UK Myeloma Research Alliance Risk Profile, were compared using retrospective (test analysis) and prospective data (validation) to assess, whether they yield similar results. Moreover, FA is already utilized to guide therapeutic management and its usefulness has been most clearly demonstrated in oncology [9, 12–16]. However, in routine clinical practice, these tools are currently assessed and considered in only about 20% of myeloma patients, and the consistent integration of FA into MM-tumorboards (MM-TBs) for therapeutic decision-making has not been evaluated [7–13].

At our Comprehensive Cancer Center Freiburg (CCCF), FA is conducted via the R-MCI. This validated MM-specific risk score can also be reliably conducted using retrospective data [13] and is consistently assessed in our MM patients before treatment initiation. Furthermore, it has been integrated into our electronic Tumorboard online system (TOS) [7, 9, 12, 13, 17]. The R-MCI web tool enables the immediate calculation of the R-MCI (www.myelomacomorbidityindex.org [18]) for physicians, study nurses and research assistants.

As FA, exemplified by the use of the R-MCI in MM-TB, and multidisciplinary care are novel standards in improving patient outcomes by finding the fine line between under- and overtreatment [7, 8, 17], the aim of this study was to determine (a) the extend and the reliability of the integration of the R-MCI in our MM-TB, (b) its impact on treatment guidance at baseline and (c) R-MCI changes during follow-up in consecutive MM patients at our center.

## Materials and methods

### Data sources and study design

We performed this exploratory study in consecutive MM patients who were presented in our MM-TB at the CCCF, as described [8, 17] All patient information was recorded in the electronic documentation system, Medoc, and retrieved from it [9, 12, 13, 17].

**Fig. 1:**
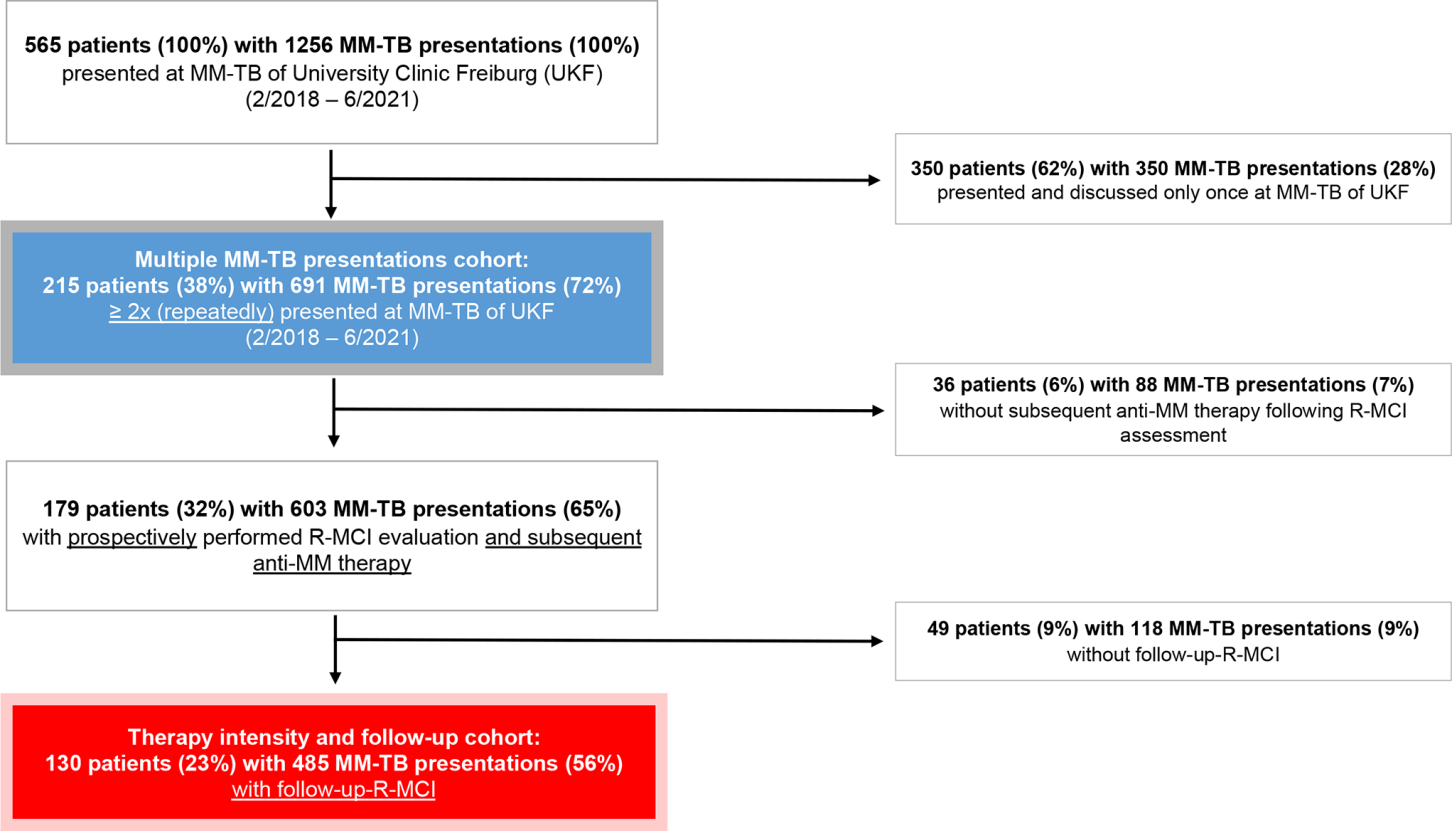
Patient disposition (Flow-Diagram)

Patient dispositions are outlined in Fig. 1: we retrieved 565 MM patients (100%) with 1256 MM-TB presentations (100%), being presented and discussed either once or repeatedly between February 2018 and June 2021. To maximize the use of prospective and unbiased data for the follow-up, we focused on patients with a minimum of two MM-TB presentations, anticipating at least two R-MCI assessments/patient (marked in light blue in Fig. 1: *n* = 215 patients). This formed a cohort of 215 MM patients with multiple TB presentations (*n* = 691), in which we evaluated the R-MCI data and number of MM-TB presentations/patient (2, 3, 4, 5, 6, 7, ≥ 8). Out of these 215 patients, 179 patients with 603 MM-TB presentations underwent prospectively performed R-MCI assessments and subsequent anti-myeloma-therapy. Follow-up R-MCI calculations were available for 130 of these patients (with 485 MM-TB presentations; marked in red in Fig. 1). This ‘follow-up cohort’ was analyzed for patient characteristics which included age, MM staging, number of therapy lines at MM-TB assessment and therapy data (induction, maintenance or subsequent MM therapy).

The aim of this study was to determine (a) the extend and the reliability of the integration of the R-MCI in our MM-TB, (b) its impact on treatment guidance at baseline [a) and b) performed in cohort of 215 MM patients with multiple TB presentations (*n* = 691), marked in light blue in Fig. 1] and (c) R-MCI changes during follow-up in consecutive MM patients at our center (*n* = 130 patients, with 485 TB presentations, marked in red in Fig. 1). Moreover, we compared therapy intensity (dose reductions done vs. not) in subgroups of patients, categorized by frailty (R-MCI: fit, intermediate-fit, frail) vs. age (< 60, 60–69, ≥ 70 years) in analogy to Holler et al. [9].

The study was performed according to the guidelines of the Declaration of Helsinki and Good Clinical Practice. All patients gave their written informed consent for institutionally initiated research studies and analyses of clinical outcome studies conforming to the institutional review board guidelines. The ethics committee of the University of Freiburg (UKF) approved the trial protocol (EV, 81/10 + 22-1491-S1).

### R-MCI assessment

The R-MCI comprises five weighted risk factors, namely renal and lung function, Karnofsky performance status (KPS), frailty and age. Additionally, it allows to include cytogenetics (CGs) if available [7]. We had tested the R-MCI in other centers and used in real-world Myriam- (https://www.iomedico.com/study/myriam/) and Caro-studies (https://www.iomedico.com/study/caro/), where pulmonary function via lung function test was substituted by anamnestic lung function assessment. Since “Lung function”-testing in the R-MCI had been repeatedly requested by reviewers - to be assessed via lung function test as a more objective measure than via GOLD criteria, smoking status or dyspnea upon exertion - it is included in the diagnostic work-up at our center (i.e. before intensive treatment, such as ASCT). If lung function testing is unavailable, ‘smoking status, its mandatory cessation before SCT/intensive treatment, no advanced GOLD criteria and no dyspnea upon exertion’ have been used as substitutes. When calculating the R-MCI using the web tool (www.myelomacomorbidityindex.org [18]), the lung function assessment can be categorized as either no/mild or moderate/severe. This assessment can be based on the patient’s medical history or specific lung function tests. Therefore, the R-MCI calculation is still possible, even if values such as forced expiratory volume (FEV1) are unavailable.

Since the R-MCI had been integrated into our TOS at our UKF center to be readily available for patient- and fitness-related questions, we assessed its extent of coverage within the MM-TB.

**Fig. 2:**
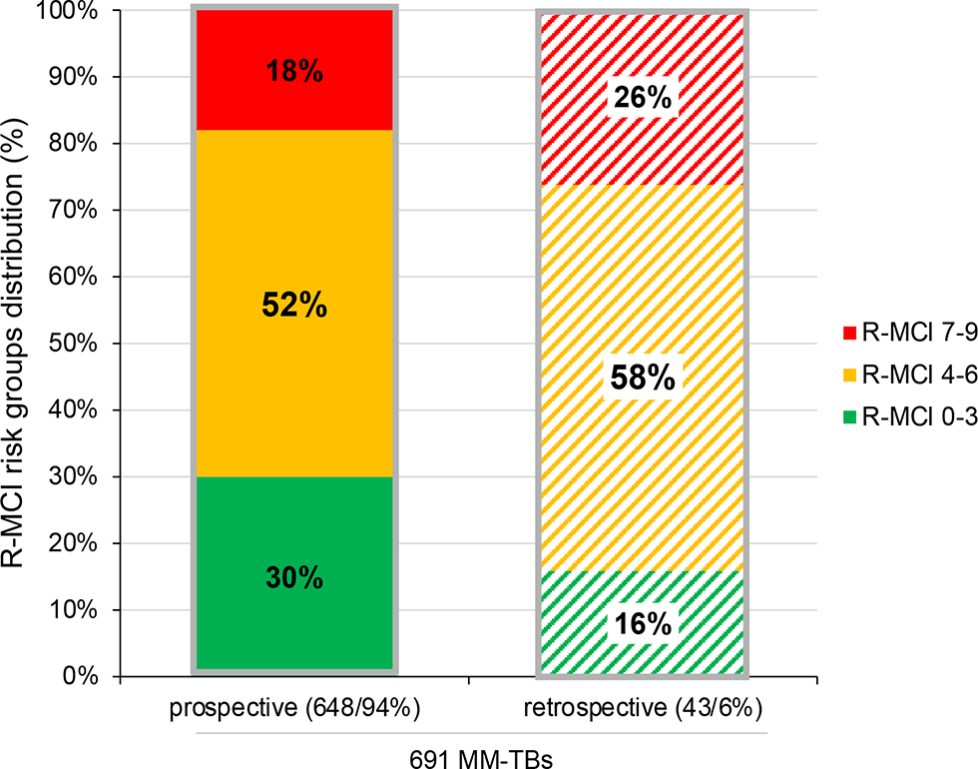
R-MCI distribution of 215 repeatedly presented MM-TB patients in 691 MM-TB presentations, prospectively vs. retrospectively scored

In cases, where the R-MCI had not been found integrated within the MM-TB (Fig. 2 and 6%), we used the online R-MCI calculator (www.myelomacomorbidityindex.org [18]) to determine the R-MCI retrospectively, as described [13].

### Dose reductions and follow-up analysis

Our aim was to examine the impact of the R-MCI on therapy decisions, analogous to prior reports. Therefore, we focused on patients who were initiated on anti-MM therapy after initial R-MCI assessment [9, 12]. We defined a shorter time span as compared to prior studies, here of a maximum of 6 months between the R-MCI assessment and subsequent therapeutic decision to assess the possible impact of R-MCI changes within shorter time frames. Dose reduction was defined according to dose reduction recommendations in UKF/CCCF therapy handbooks and chemotherapy schedules as described [9]. Decisions of dose reductions were based on physicians’ choices [9, 12], but these were here influenced by TOS-integrated R-MCI data. This was different to previous data by Holler et al., where the R-MCI had not been obligatory to insert into our TOS-MM-TB [9].

**Fig. 3:**
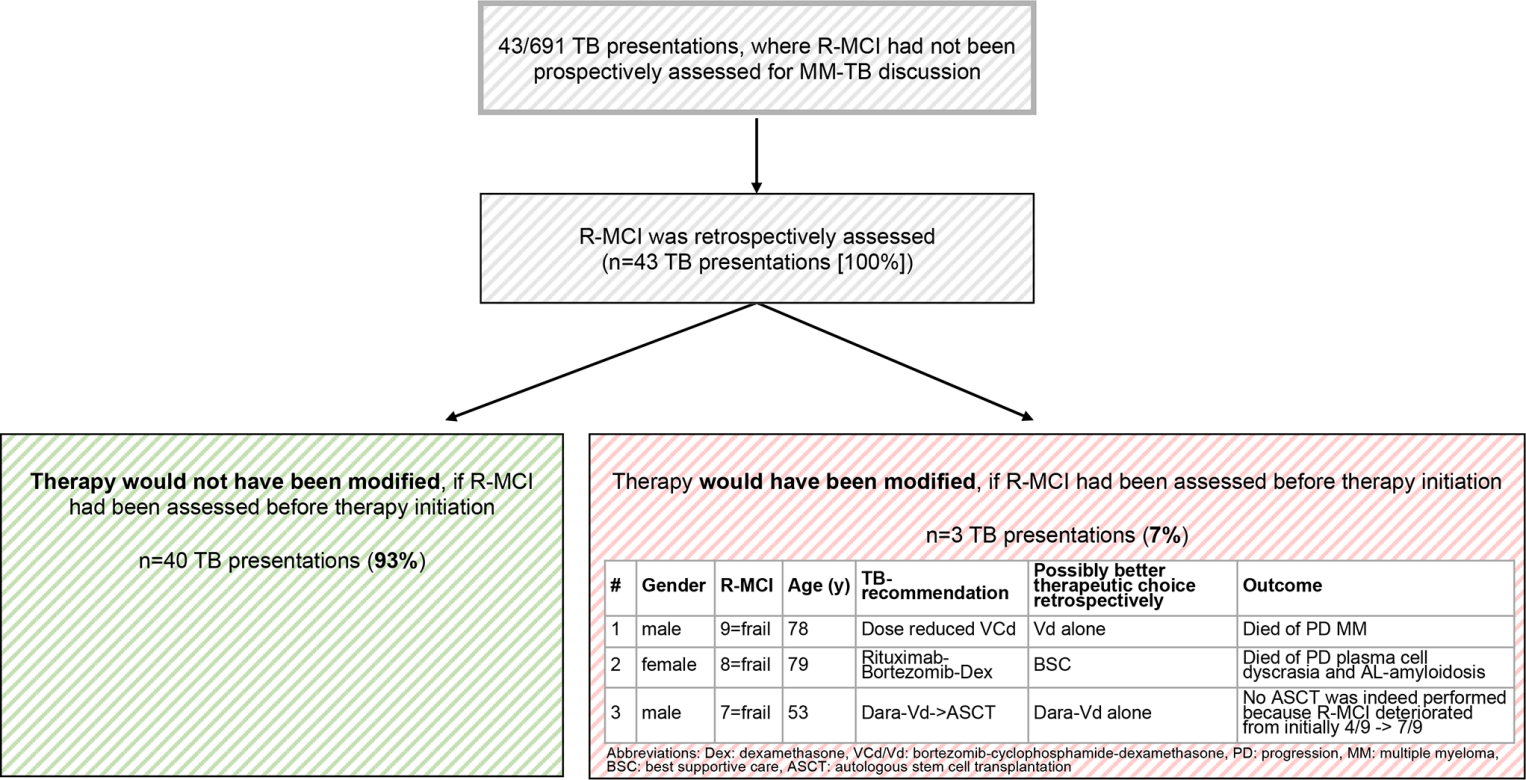
Clinical reassessment in MM patients, where R-MCI had not been scored prospectively, but was retrospectively assessed

In 43 TB presentations (6%), the R-MCI had not been prospectively inserted into our TOS-MM-TB, thus was therein unassessed for the MM-TB discussion. In these cases, two physicians (ED + ME), both trained in onco-geriatrics, independently determined, whether chosen MM-decisions and given therapy suggestion would have been different, if the R-MCI had been assessed before therapy initiation (Fig. 3). Through these data, we could determine, whether therapy intensity aligned with the fitness levels determined via R-MCI, providing full dose for fit patients and reduced intensity for frail patients.

**Fig. 4:**
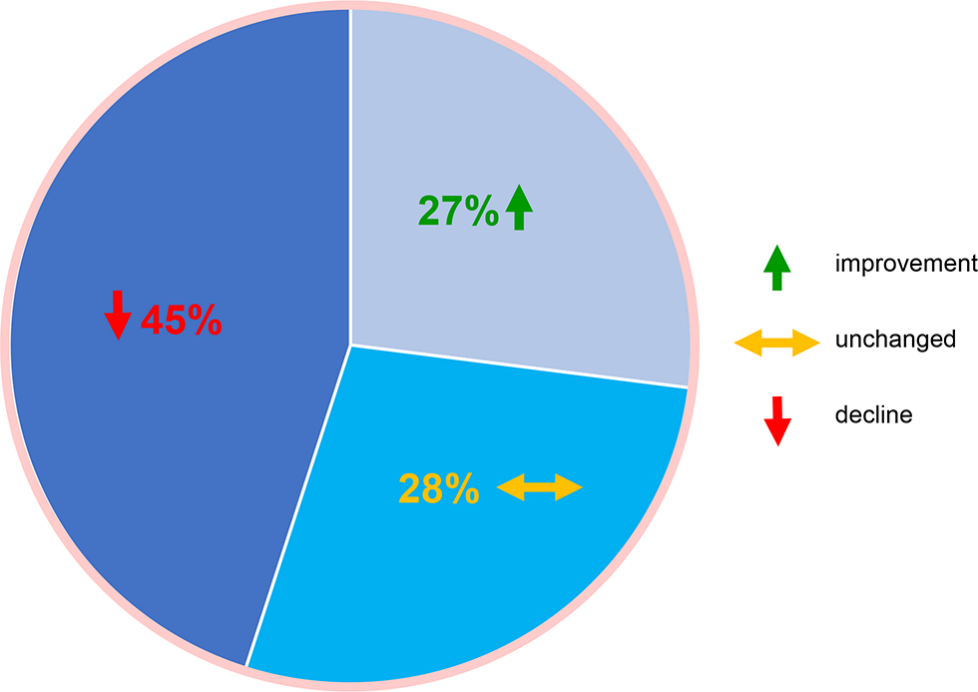
Comparison of R-MCI scores at initial vs. follow-up assessment (*n* = 130 patients with 485 MM-TBs)

To further assess the therapy decisions, we examined whether patients’ constitution had changed in a follow-up analysis. Therefore, we evaluated the specific distribution of patients among R-MCI scores before start of therapy (T0) and follow-up (T1; Table 1). We also assessed the R-MCI changes over time, namely whether the R-MCI improved, remained unchanged or deteriorated (Fig. 4), in line with previous studies [9, 12].

**Table 1:** Comparison of R-MCI at T0 versus T1 with number of patients and Mean / median (range) values

*R*-MCI subgroups	R-MCI 0–9 (*n* = 130)	T0 = initial assessment (# of patients)	T1 = follow-up assessment (# of patients)
	0	2	2
	1	6	2
Fit	2	18	10
	3	19 (0–3: *n* = 45 = 34.6%)	21 (0–3: *n* = 35 = 27%)
	4	26	27
Intermediate-fit	5	24	27
	6	12 (4–6: *n* = 62 = 47.7%)	19 (4–6: *n* = 73 = 56%)
	7	16	15
Frail	8	7	6
	9	0 (7–9: *n* = 23 = 17.7%)	1 (7–9: *n* = 22 = 17%)
	***Mean / Median (range)***	***4.3 / 4 (0–8)***	***4.7 / 5 (0–9)***

*Abbreviations* T0: initial R-MCI assessment before treatment initiation, T1: follow-up assessment of potential R-MCI changes after treatment and median follow-up of 5 months

### Statistical analysis

The trial was analyzed descriptively, providing means, median and range for continuous measures, and absolute and relative frequencies for categorical variables. The R-MCI was assessed and compared in age subgroups of < 60-, 60-69- and ≥ 70-year-old patients, in analogy to prior analyses [9, 12] (Tables 2 and 3), as well as at initial and subsequent (= follow-up) assessment (Table 1). Data were assessed using GraphPadPrism V5.03 and SAS 9.4 (SAS Institute Inc. USA) and were analyzed as of 1/2023.

**Table 2:** Distribution of age subgroups and within their group the number and proportion fit, intermediate-fit and frail patients via R-MCI (*n* = 130)

Age subgroups	Entire group	*R*-MCI subgroup		
		Fit	Intermediate-fit	Frail
<60 years (%)	44 (34)	28 (64)	15 (34)	1 (2)
60–69 years (%)	39 (30)	13 (33)	21 (54)	5 (13)
≥70 years (%)	47 (36)	4 (9)	26 (55)	17 (36)

**Table 3:** Patient- and therapy-relevant differences in entire and R-MCI- vs. age subgroups in frequencies, age and treatment performed without (w/o) vs. with (w) dose reduction

	Entire group	*R*-MCI subgroups			Age subgroups		
		Fit	Intermediate	Frail	< 60 years	60–69 years	≥ 70 years
Patients (%)	130 (100)	45 (34)	62 (48)	23 (18)	44 (34)	39 (30)	47 (36)
Median age (range)	66 (38–86)	58 (38–78)	68 (48–84)	77 (72–86)	55 (38–59)	65 (60–69)	77 (70–86)
Patients w/o dose reduction (%) / w dose reduction (%)	58 (45) / 72 (55)	28 (62) / 17 (38)	24 (39) / 36 (61)	6 (26) / 17 (74)	24 (55) / 20 (45)	20 (51) / 19 (49)	14 (30) / 33 (70)

*Abbreviations* intermediate: intermediate-fit, w/o: without, w: with

## Results

### Number of MM-TB presentations per patient

**Fig. 5:**
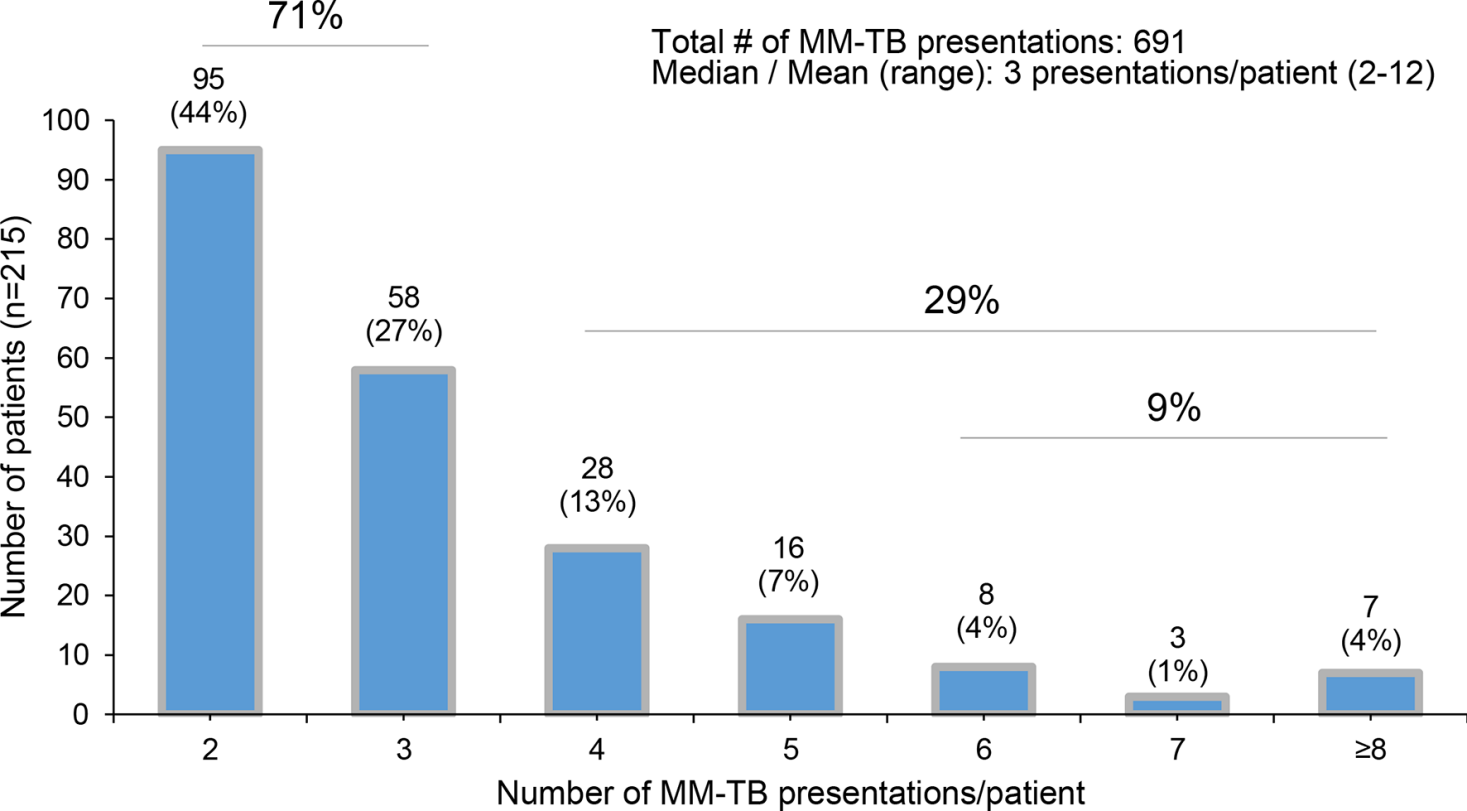
Number of MM-TB presentations *≥* 2, allowing follow-up assessments as the requisite of study inclusion (*n* = 691 MM-TBs)

For patients with ≥ 2 MM-TB presentations (*n* = 215, Fig. 1), we assessed the median TB-presentations/patient, which were three within our 3-year assessment period (2/2018–6/2021), in line with prior analyses of our group (Fig. 5) [8, 17, 19].

Figure 5 displays the number and frequencies of patients with repeated (2, 3, 4, 5, 6, 7 or ≥ 8) TB presentations. Within the 3-year study, most patients were presented within our MM-TB 2 or 3 times (71%), ≥ 4 presentations were found in 29% of patients and ≥ 6 presentations/patient were rare (9%, Fig. 5).

### R-MCI adherence at the MM-TB and prospectively vs. retrospectively assessed R-MCI

Within 691 MM-TBs (Figs. 1 and 5), prospective R-MCI scores were available in 94% (648/691 MM-TB protocols, Fig. 2). In the remaining 6% of presentations (*n* = 43), the R-MCI was calculated retrospectively.

Regarding the R-MCI subgroups of the prospectively assessed scores, 30% patients were classified as fit (R-MCI 0–3), 52% as intermediate-fit (R-MCI 5–6) and 18% as frail (R-MCI 7–9) in line with prior analyses (Fig. 2, left column) [8, 9, 12, 17]. In the few retrospectively scored presentations, fit, intermediate and frail patients were observed in 16%, 58% and 26%, respectively (Fig. 2, right column). Thus, patients with non-included R-MCI assessment within the MM-TB TOS seemed 1.9-fold less fit (30%→16%) and 1.1-fold more intermediate-fit (52%%→58%) or 1.4-fold frailer (18%→26%) as compared to the prospectively evaluated cohort (Fig. 2).

### Differences in therapy decisions without prospective R-MCI assessment

In 43 MM-TB presentations (6%), the R-MCI had not been utilized to aid the TB decision (Fig. 2). In 40 cases (93%), the MM-TB decisions (reassessed by ED + ME) were not found to have been chosen differently (Fig. 3). However, in three patients, both physicians (ED + ME) would have modified treatment according to the R-MCI. Notably, all three were frail according to the R-MCI, including the only < 60-year-old patient with an R-MCI of 7/9.

The first 78-year-old male patient had received dose-reduced bortezomib-cyclophosphamide-dexamethasone (VCd), although, due to his R-MCI of 9/9 (= utmost frailty), Vd might have been more appropriate retrospectively. The second 79-year-old female patient with an R-MCI of 8/9 had received rituximab-bortezomib-dexamethasone treatment due to high CD20 plasma cell expression, where best supportive care (BSC) would have been more appropriate retrospectively. The third 53-year old male patient with an R-MCI of 7/9 had received Daratumumab-Vd (DVd, adapted according to the Alcyone study [20]) and was initially intended to undergo ASCT due to his age. Retrospectively, the therapeutic choice would have been DVd alone. Thus, the outcome (progressive disease in 3/3, subsequent hospitalization in 3/3 and substantial dose reduction being necessary in 2/3 and BSC in 1/3) of all three frail patients suggested that better therapy choices could have been made, if the R-MCI had been utilized to aid in the therapeutic process. Notably, MM-related death occurred in 2/3 patients.

### Patient characteristics

We focused on 130 patients with *≥* 2 MM-TB presentations/patient (with 485 MM-TB presentations), subsequent anti-MM treatment and R-MCI follow-up assessment (Fig. 1; Table 4). Their median age of 66 years (range 38–86) at initial presentation was typical for referral and university centers, with 62% being male and 38% female. Expectedly, the most common paraprotein types were IgG and kappa light-chains in 56% and 71%, respectively (Table 4). Advanced ISS stage (2 or 3) was frequent with 68%. During the observation period, a median of two therapy lines/patient (range 1–9) had been performed, with induction, maintenance, and later-line (relapse) therapy being performed in 31%, 17% and 52%, respectively.

### Age subgroups and further analysis of the distribution of R-MCI scores within their groups

Age subgroups of patients < 60, 60–69 and ≥ 70 years were equally distributed with 34%, 30% and 36%, respectively (Table 2). Expectedly, younger patients aged < 60-years were mostly fit (64%) or intermediate-fit (34%), while frail patients were rare (2%). In the group of 60-69-year-old patients, a substantial shift from fewer fit (33%) to more intermediate-fit (54%) and frail patients (13%) was noted. Even more strikingly, however, 9% of ≥ 70-years-old patients were classified as fit, whereas most (55%) were intermediate-fit, and 36% were frail (Table 2). Thus, being fit was 4.5-times more prevalent in elderly patients than frailty in young patients.

### Patient- and therapy-relevant differences in entire cohort and R-MCI and age subgroups

Differences in patient frequencies, median age and dose reduction in the entire patient group, as well as in R-MCI vs. age subgroups are summarized in Table 3. While dose reduction in the entire patient group occurred frequently (55%), this was less common in R-MCI fit patients (38%), and increased in intermediate-fit and frail patients to 61% and 74%, respectively.

**Table 4:** Characteristics of actively treated MM patients with follow-up R-MCI assessment (*n* = 130)

Variables	Number of patients (%)	Median (range)
**Age at therapy initiation**		66 (38–86)
**Males : females**	81 (62) : 49 (38)	
**MM type**		
IgG / IgA / IgM / biclonal / LC only	73 (56) / 20 (15) / 2 (2) / 2 (2) / 33 (25)	
Kappa / lambda / biclonal	92 (71) / 36 (28) / 2 (1)	
AL-Amyloidosis	3 (2)	
**ISS stage**		
I / II / III	39 (32) / 37 (30) / 47 (38)	
**Therapy lines @ MM-TB assessment**		2 (1–9)
**Induction (first-line) MM therapy**	40 (31)	
**Under maintenance therapy**	22 (17)	
**Subsequent (later line) MM therapy**	68 (52)	

*Abbreviations* MM: multiple myeloma; ISS: international staging system; @: at

Notably, dose reduction in < 60-, 60-69- and ≥ 70-year-old patients were performed in 45%, 49% and 70%, respectively. Thus, dose reduction was less common in fit patients than in < 60-year-old patients. Conversely, in intermediate-fit and frail patients, dose reduction occurred more frequently than in 60-69- and ≥ 70-year-old patients (Table 3).

### R-MCI changes before start of therapy (T0) vs. follow-up (T1; serial R-MCI assessment)

Serial R-MCI assessments were available for 130 patients within 485 MM-TB presentations and with anti-MM therapy being performed (Fig. 1). Our follow-up assessment of potential R-MCI changes was here conducted earlier than in previous analyses (after a median of 5 rather than previously after 11 months) [9, 12]. The mean and median R-MCI at T0 were 4.3 and 4 (intermediate-fit) and at T1 4.7 and 5 (remaining intermediate-fit), respectively (Table 1). The precise distribution of patients among R-MCI scores at T0 and T1 is displayed in Table 1. Notably, over the median follow-up period of 5 months (range: 0–25), the number of fit patients slightly decreased (T0: 34.6% → T1: 27%), while the number of intermediate-fit patients increased (T0: 47.7% → T1: 56%), and the number of frail patients remained stable (T0: 17.7% → T1: 17%).

Additionally, we assessed in all patients, whether their constitution via R-MCI changed within our follow-up period: in 55%, this improved or remained unchanged, whereas in 45%, a decline was noted (Fig. 4).

### Dose reduction in relation to number of MM-TB presentations

**Fig. 6:**
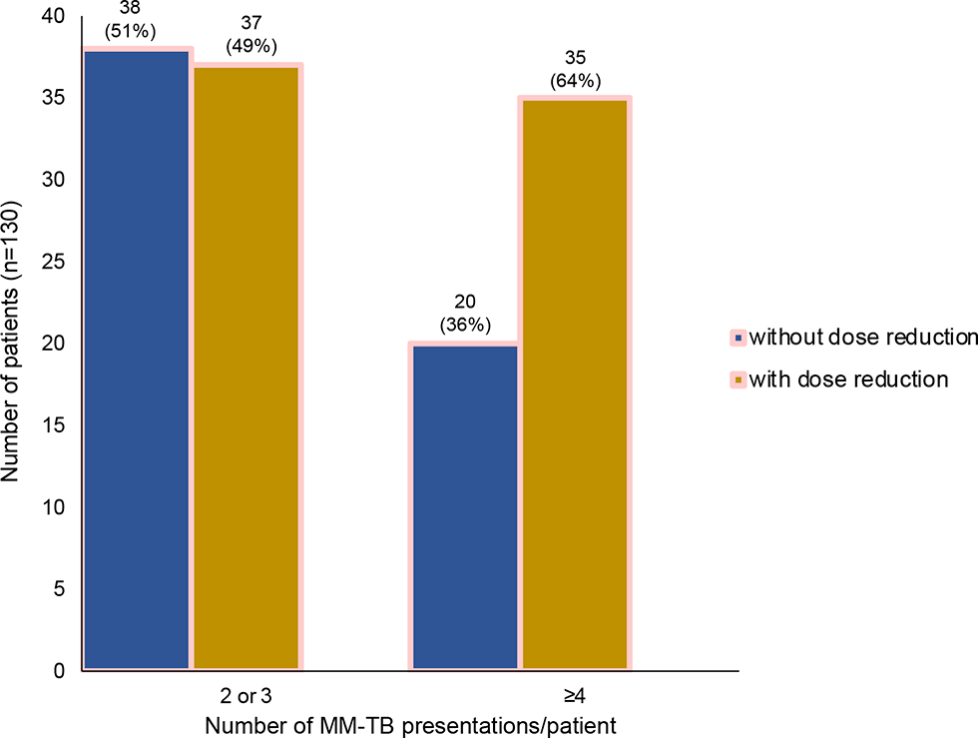
Comparison of patients without dose reduction (blue) vs. with dose reduction (yellow) in relation to few (≥ 3) or more (≥ 4) TBs (*n* = 130 patients in 485 MM-TBs)

In terms of dose reduction, we also analyzed, whether frequencies of dose reduction were related to the number of TB presentations/patient, namely, whether with lesser vs. more frequent TB discussions, dose-reductions increased due to MM patients’ quality of life (QoL) decreasing with subsequent treatment lines, as described previously (Fig. 6) [21].

In patients with 2 or 3 TB-presentations, no dose reduction vs. dose reduction were performed in similar frequencies with 51% and 49%, respectively. This was different in patients with more advanced or difficult-to-treat MM showing ≥ 4 TB-presentations: no dose reduction was here performed in only 36%, whereas dose reduction was much more frequent in 64% (Fig. 6). The frequencies of no dose reduction vs. dose reduction in each subsequent 2 to ≥ 8 TB frequencies are outlined in Suppl. Figure 1.

## Discussion

Since therapy decision-making is ideally supported by FA to individualize treatment especially for frail patients [14], we integrated the R-MCI into our MM-TB [8]. This integration allows all users to readily distinguish fit from frail patients and expeditiously adjust therapy intensity during our weekly performed interdisciplinary MM-TB discussion [8, 9, 12, 13] While various functional tests, including different comorbidity scores and geriatric functional tests have been examined [12, 22–24], the R-MCI was explicitly helpful to individualize treatment decisions and improving the tolerance of MM therapy [9]. Additionally, the R-MCI has been recognized as the only comorbidity index that did not show significant differences in risk group distribution for both retrospective and prospective data, thus it was reliably assessable from both data sets [13]. Another convenience is the user-friendly R-MCI homepage (www.myelomacomorbidityindex.org [18]).

While fitness assessments are used for tailoring therapy in other hematological diseases, these are not routinely established in MM patients, despite studies aiming to elucidate their usefulness [9, 12, 15, 16, 25]. FA have been described as challenging to integrate into everyday clinics due to time constraints [12, 22–24]. The pivotal outcome of our study was the successful integration of the R-MCI into MM-TB protocols in > 90%, confirming previous studies of the R-MCI being resourceful [7–9, 12, 13, 23, 26]. The establishment of the R-MCI in our MM-TB exemplified that frailty scores can be readily used in everyday clinics. Albeit the R-MCI calculation is specifically requested in our TOS MM-TB system, its use and integration for each patient therein was not obligatory within TOS during the assessment period, therefore the availability in > 90% of our large MM-TB cohort (*n* = 691 MM-TB cases; Fig. 1) was a success. Of note, the R-MCI was unavailable in 43 patients (6%) only, who had been externally referred to our UKF/CCCF: either missing data were reasons for its non-use, or physicians from different disciplines/departments were overriding the TOS-integrated R-MCI scoring. In these patients, the subjective assessment of the introducing physicians within the MM-TB sessions was utilized (which would have otherwise been complemented by the R-MCI). Meanwhile, we have improved the TOS MM-TB software to make it mandatory to enter the R-MCI parameters required for its calculation and thereby ensuring that R-MCI scores are available in 100% of patients. Consistent with previous analyses, we affirmed the rarity (2%) of frailty in < 60-year-old patients but acknowledged its significance. This was indeed important to determine, because in the only frail 53-year-old patient, CD38-based induction and ASCT had been the TB-recommendation, whereas CD38-based therapy alone would have been the better TB-choice according to the R-MCI assessment. We observed an increase in frailty to 36% in ≥ 70-year-old patients, albeit among them also 9% were fit and 55% were intermediate-fit. Dose reduction was performed in alignment with fitness levels in 38% in fit, 61% in intermediate-fit and 74% in frail patients. Decision-making without an available R-MCI revealed that in three frail patients, therapeutic decision could have been facilitated with exact knowledge of patients’ fitness. Consequently, therapy choices would have differed, if the R-MCI had been assessed before therapy initiation. Thus, in MM treatment, it is not just about finding the most innovative therapy regimen but, equally importantly, about finding the most fitting therapy intensity. These three cases showed that by implementing the R-MCI in the therapy decision, we would not have chosen a more innovative therapy, but rather a less intense therapy dose. Very recent therapy options (i.e. CAR-T-cells, BITEs and ADC) might also be of interest to illustrate in an updated analysis, albeit one may already suspect from the current data, that upon fitness misjudging, treatment adjustments remain pertinent.

Of note in this study and different to others [9, 12] was, that our follow-up assessment of potential R-MCI changes was conducted earlier, after a median of 5 months, and that the R-MCI showed fitness improvement or stability in 55%, while a decline was observed in 45% of cases. In previous studies, where the follow-up was conducted after a median of 11 months, the R-MCI showed rather an improvement in 90% of patients than decline in only 10%. This indicates that it may be more advisable to evaluate changes in fitness and QoL after a longer period following the initiation of therapy, in order to prevent temporary therapy side effects from affecting QoL. Therefore, to possibly better capture MM patients’ advances in performance, therapy endurance and QoL, a defined latency of approximately 1 year appears more suitable than < 6 months. Likewise, the common practice of repeatedly assessing QoL domains (i.e. in QoL questionnaires in clinical studies) over very short periods may be less practical in view of our data [9, 12, 21, 25, 27].

Our assessment of the MM-TB over a 3-year period revealed that 38% of patients were presented at least twice, resulting in a total of 691 MM-TB presentations. At our center, all MM patients with newly diagnosed and relapsed disease are discussed in our weekly MM-TB, including demanding cases, such as plasma cell leukemia, extramedullary cases or triple-class (to penta-) refractory patients. Specifically, regarding our study, the patients we analysed were, on median, at the second line of therapy. Only 31% of them were undergoing induction therapy, while 52% were receiving later-line MM therapy, indicating that most of thems had relapsed/refractory disease. Moreover, the median number of MM-TB presentations for our patients was 3 during the investigation period. These repeated presentations indicate that the MM treatment was fairly demanding and required interdisciplinary discussion. Thus, among these patients, 71% had 2–3 MM-TB presentations within our observation period, while 29% had ≥ 4 presentations, and 9% had ≥ 6, supporting our view on very frequent TBs. MM-TB outcome data of 2020/21- and 2012-2014-analyses as well as our data here seem valuable, as we had previously described the postulated survival benefit in patients with ≥ 3 TB discussions as error-prone, occurring due to an immortal time bias (since patients need to survive long enough to be discussed more often). Therefore, time-biased results should not lead to the conclusion that more TBs will increase patients’ survival. Instead, insightful discussions within one or few meaningful, long-lasting TBs, ideally in interdisciplinary teams, will generate most profound results for cancer patients [19, 28].

Of interest was, that the number of dose-reduced therapies in our entire cohort was higher than in our previous study (55% vs. 41% [9], respectively). This difference could be related to our focus on MM-TB patients, whereas Holler et al. assessed physician-based therapeutic decisions for consecutive MM patients [9]. Notably, in the study by Holler et al., FA was conducted retrospectively rather than before treatment. This finding supports a prior study which found that the percentage of patients who started less-intensive therapy was higher when FA was conducted before treatment compared to when it was not performed [15]. Our results revealed that dose adaptions were regularly performed in frail patients. Although age-based dose reduction was also observed, they seemed more error-prone than if the R-MCI was included in the decision-making: older patients (≥ 70 years) were treated with dose reduction in 70%, albeit only 36% of them were frail, while 9% were fit and 55% were intermediate-fit. Consequently, this group of patients was rather undertreated in substantial numbers, as previously described [7]. In line, younger patients can be frail and are important to decipher likewise. Therefore, the assumption that younger patients should always receive full-dose treatment, and elderly patients should not, may often be error-prone. This confirms that age alone is not sufficient to determine patients’ health status and therapy endurance. Instead, FA is superior in specifying patients’ constitution and biological age [7–13, 26] which is why treatment decisions should rather depend on FA tools, such as R-MCI or others.

Strengths of our analysis included the precise examination of a large MM-TB cohort, the R-MCI as a uniform screening tool and its repeated analysis, and the observed dose reduction, well-associated with R-MCI-, rather than age-subgroups. Our observation period of 3 years was substantial, and the detailed examination of patients with various numbers of TB presentations and deterioration in their health status (R-MCI) exhibited their complexity. This is in line with a prior analysis in MM patients with first- vs. later lines of therapy [21], which, however, did not assess QoL changes in consecutive but different follow-up cohorts. Our analysis was well comparable to prior analyses [8, 17, 19] and revealed the quality of one out of 24 TBs at our UKF/CCCF: while the quality results of our MM-TB, the decisions, pathway- and guideline-adherence, TB-compliance, referrer satisfaction, and improvement of clinical trial inclusion have been described previously, our data here proved that FA integrated into TBs is feasible and does support therapy decisions.

Limitations of the study were the single-institution approach and the range of patients’ ages (38–86 years), with 64% of patients being < 70 years old. This is, however, typical for MM patients in tertiary centers and suggests that our data are even more relevant in older patients. Additionally, the specific conditions for our study inclusion (MM-TB presentations ≥ 2, subsequent therapy being instituted, and performed follow-up R-MCI) focused on more complex MM patients. Our cohort did include patients receiving different treatment with induction, maintenance and later-line treatment, since our aim was not survival analyses, rather than to assess the completeness of the R-MCI within our MM-TB, whether therapy decisions in terms of fitness vs. age cohorts were different and how dose-reductions were performed. The aim of this study was therefore not to determine exact therapy choices of newly diagnosed MM vs. relapsed/refractory MM, or within different therapy lines, nor whether patients did profit from dose-reductions, because we and others had shown this in prior publications [7, 9, 10, 29–39]. Moreover, our follow-up assessment was conducted within a relatively short period which should ideally be performed after ~ 1 year, according to Scheubeck and Holler [9, 12]. Another criticism may arise from the retrospective evaluation of three patients (whose R-MCI was unavailable in TOS and who received different, more intensive treatment due to the unavailability of their R-MCI). This introduced bias as their outcome was known during our reassessment of therapy choices. Last, since we had examined survival repeatedly in similar MM cohorts, via R-MCI and age subgroups and in different MM-TB cohorts [7–9, 12, 13, 17], this was not repeated here.

In conclusion, our results demonstrate the widespread use of the R-MCI within the MM-TB. Further research through prospective clinical trials seems essential to determine optimal, personalized treatment options for each patient. Building on this approach, Mian et al. published a systematic review including 43 clinical trials considering frailty tools and showed an encouraging trend to incorporate frailty assessments in clinical evaluations and treatment decisions [10], in line with our data of the R-MCI-integration in TOS-MM-TBs in > 90%. More patients were treated with dose reduction when FA was performed before therapy, compared to data of our previous study [9] and the association between the R-MCI and chosen therapy intensity was better than via age cohorts, which further underlines that the R-MCI is a more precise predictor than age alone. We could show that the recommendations to establish therapy decisions on FA can be directly implemented in TBs. Therapy decisions for intermediate-fit patients appear more complex than for fit and frail patients, because some intermediate-fit patients may endure full-dose treatment, while others need dose-reduction. Therefore, this group of patients should be analyzed further. Today, some MM experts distinguish only two groups of fit vs. frail patients [6, 9, 35]. Prospective studies using the R-MCI as an important tool for therapeutic decision-making are in process at our CCCF. Most importantly, these and other important TB analyses have led to our better interpretation of cancer care, in close collaboration with statisticians [17, 40, 41], which is essential to produce reliable evidence for future progress. We are grateful that these productive collaborations continue to exist at our and other CCCs.

## Electronic supplementary material

Below is the link to the electronic supplementary material.

Supplementary Material 1

## Data Availability

The data that support the findings of this study are available from the corresponding author (ME) upon reasonable request.
