# Supplementary material for: Optimizing individualized therapy decision-making in multiple myeloma (MM): integration and impact of the Revised Myeloma Comorbidity Index in the MM-tumor board
Source: Ann Hematol. 2024 Sep 21;104(1):593–603. doi: 10.1007/s00277-024-06010-5 (PMC11868216; doi:10.1007/s00277-024-06010-5)
Supplement: Supplementary file 1 — Supplementary Material 1 [file 277_2024_6010_MOESM1_ESM.docx]

**Supplementary Fig. 1.** Comparison of patients without dose reduction (blue) vs. with dose reduction (yellow) in 2 to ≥ 8 MM-TBs (n = 130 patients in 485 MM-TBs)
